# Clinical outcome of surgical management for symptomatic metastatic spinal cord compression from prostate cancer

**DOI:** 10.1186/s12894-020-00713-3

**Published:** 2020-09-05

**Authors:** Yasuhide Miyoshi, Takashi Kawahara, Masahiro Yao, Hiroji Uemura

**Affiliations:** 1 Department of Urology and Renal Transplantation, Yokohama City University Medical Center, 4-57 Urafune-cho, Minami-ku, Yokohama, Kanagawa 2320024 Japan; 2 Department of Urology, Yokohama City University School of Medicine, 3-9 Fukuura, Kanazawa-ku, Yokohama, Kanagawa 2360004 Japan

**Keywords:** Prostate cancer, Castration-resistant prostate cancer, Spinal cord compression, Bone metastas

## Abstract

**Background:**

Metastatic spinal cord compression (MSCC) from prostate cancer (PC) influences not only patients’ prognosis but also their quality of life. However, little is known about the clinical outcome of surgery for MSCC from PC. We evaluated both the oncological and functional outcomes of decompression and reconstruction surgery for patients with symptomatic MSCC from PC.

**Methods:**

We assessed 19 patients who underwent decompression and reconstruction surgery for symptomatic MSCC from PC. Of these 19 patients, 8 had metastatic hormone-naïve PC (mHNPC) and 11 had metastatic castration-resistant PC (mCRPC).

**Results:**

The median age of the patients with mHNPC and mCRPC was 72 and 65 years, respectively. The median prostate-specific antigen level at the time of diagnosis of MSCC in patients with mHNPC and mCRPC was 910 and 67 ng/mL, respectively. Although two of eight patients (25.0%) with mHNPC were ambulatory preoperatively, six patients (75.0%) were ambulatory postoperatively. Among 11 patients with mCRPC, only 3 (27.3%) were ambulatory preoperatively, while 6 (54.5%) were ambulatory postoperatively. The median postoperative overall survival among patients with mHNPC and mCRPC were not reached and 8 months, respectively.

**Conclusions:**

Decompression and reconstruction surgery for symptomatic MSCC from PC might contribute to a favorable functional outcome among men with mHNPC and mCRPC. However, its role in improving the oncological outcome remains unclear. The treatment strategy should be chosen by shared decision-making among patients, urologists, radiation oncologists, and orthopedic surgeons.

## Background

The incidence of prostate cancer (PC) has increased worldwide [1]. Although prostate-specific antigen screening has contributed to improvement in PC-related mortality [2], PC remains a leading cause of mortality and morbidity worldwide [1]. Metastatic hormone-naïve PC (mHNPC) is androgen-dependent, and androgen ablation therapy is initially effective; however, most patients with mHNPC become resistant to androgen ablation therapy and failed to metastatic castration-resistant PC (mCRPC) [3]. Osseous metastases are common in both patients with mHNPC and mCRPC [4, 5] and impair patients’ quality of life because of skeletal-related events. Approximately one-third of PC metastases to the spine become symptomatic, resulting in metastatic spinal cord compression (MSCC) [6] or mechanical instability [7] .

MSCC occurs in 5% of patients who die of cancer [8] and contributes to an unfavorable prognosis and poor quality of life. MSCC can cause irreversible neurological impairment and a short survival time; therefore, an effective treatment strategy for MSCC is important to not only prolong the survival time but also improve the quality of life among men with mHNPC and mCRPC [9].

Decompression surgery is the standard of care for symptomatic MSCC from various cancers, including PC [10]. However, the clinical outcome of surgery for MSCC from PC has not been fully described because of the limited number of patients. In this study, we evaluated the oncological and functional outcomes of decompression and reconstruction surgery for MSCC from PC.

## Methods

### Patients

We retrospectively assessed 19 patients who underwent decompression and reconstruction surgery for symptomatic MSCC from PC from 2002 to 2017 in Yokohama City University Medical Center and Yokohama City University Hospital. The indication for surgery was determined by multidisciplinary team management (shared decision-making among the patients, urologists, orthopedic surgeons, and radiation oncologist) under consideration of each patient’s prognosis, neurological deficits, and overall health status. In general, patients with a < 1-year prognosis and fixed neurological deficits were not recommended for surgery. The metastatic site and spinal cord compression were evaluated by computed tomography and magnetic resonance imaging. Before surgery for MSCC, all patients received high-dose dexamethasone as the initial treatment to prevent irreversible neurological impairment. Of the 19 patients included in the study, 8 had mHNPC and 11 had mCRPC. All patients had pathologically confirmed prostate adenocarcinoma. Clinical data were collected from each patient’s medical records. Tumor grades were classified by the Gleason grading system according to the 2014 International Society of Urological Pathology consensus [11].

The extent of disease on the initial bone scan [12] was used for objective semi-quantitative classification of the osseous metastases: 0 = normal or abnormal because of benign bone disease; 1 = fewer than 6 bony metastases, each of which is less than 50% of the size of a vertebral body (1 lesion about the size of a vertebral body was counted as 2 lesions); 2 = 6 to 20 bone metastases, sized as described above; 3 = more than 20 metastases but fewer than the number of metastases seen in a “superscan”; and 4 = “superscan” or its equivalent (i.e., more than 75% of the ribs, vertebrae, and pelvic bones). The surgical sites for MSCC were classified into five sites: the cervical spine, cervical-thoracic spine, thoracic spine, thoracic-lumbar spine, and lumbar spine.

### Surgical procedures and postoperative radiation therapies for MSCC

The standard surgical procedures were posterior decompression and stabilization. Patients without mechanical instability and with preservation of sagittal alignment of the spine were treated by decompression alone, although the surgical procedures performed were at each surgeon’s discretion. An early rehabilitation program specific to the patient’s spinal cord injury was implemented. Postoperative radiation was not used in patients with mHNPC because primary androgen deprivation therapy was expected to be effective. In contrast, postoperative radiation was recommended for patients with mCRPC if the patient could tolerate radiation therapy.

### Medical treatments for PC

The standard medical therapy for mHNPC was androgen deprivation therapy. No patients received cytotoxic agents or new androgen receptor-targeted therapy (abiraterone and/or enzalutamide) for mHNPC. After failed to CRPC, bisphosphonates or denosumab, new androgen receptor-targeted therapy, radium-223, or cytotoxic agents (docetaxel with steroids and/or cabazitaxel with steroids) were used if these agents were approved in Japan when the physician decided to use them. The treatment sequence for mCRPC was at the physician’s discretion.

### Evaluation of functional outcome

The Frankel grading classification [13] was used for the functional evaluation preoperatively and 3 months postoperatively. The Frankel grading classification reveals the extent of the neurological/functional deficit caused by spinal cord injury and was established by Frankel et al. [13] in 1969. This classification system is divided into five grades: (A) no function, (B) sensory only, (C) some sensory and motor preservation, (D) useful motor function, and (E) normal. Frankel grades D and E indicate an ambulatory state.

### Evaluation of oncological outcome

The Kaplan–Meier product-limit method was used to estimate the overall survival (OS) distribution after surgery for MSCC. All analyses were conducted using IBM SPSS Statistics software for Windows, version 24 (IBM Corp., Armonk, NY, USA).

### Ethics

The experimental procedures were conducted in accordance with the ethical standards of the Helsinki Declaration.

## Results

### Patients’ characteristics

Table 1 shows the patients’ characteristics. The median age of the patients with mHNPC and mCRPC at the time of surgery for MSCC was 72 and 65 years, respectively. The median prostate-specific antigen concentration at the diagnosis of MSCC in patients with mHNPC and mCRPC was 910 and 67 ng/mL, respectively. The sites of decompression surgery in patients with mHNPC were the thoracic spine in six (75.0%) patients and the lumbar spine in two (25.0%), and those in patients with mCRPC were the cervical spine in one (9.1%) patient, the cervical-thoracic spine in two (18.2%), the thoracic spine in six (54.5%), the thoracic-lumbar spine in one (9.1%), and the lumbar spine in one (9.1%). The median time to decompression surgery from symptom onset was 4 days in patients with mHNPC and 2 days in patients with mCRPC. Bone-targeted agents such as zoledronic acid and denosumab were used in four (36.3%) patients with mCRPC and no patients with mHNPC. Other variables at the initial diagnosis of PC are also listed in Table 1.

**Table 1:** Patients’ characteristics

	Patients with mHNPC (*n* = 8)	Patients with mCRPC (*n* = 11)
Variables at initial diagnosis of prostate cancer		
Median prostate-specific antigen (range), ng/mL	910 (98–8900)	232 (6–4271)
Biopsy Gleason scores, n (%)		
≤ 7	0 (0.0%)	2 (18.2%)
8–10	5 (62.5%)	9 (81.8%)
Unknown	3 (37.5%)	0 (0.0%)
Extent of disease on bone scan		
0	0 (0.0%)	4 (36.4%)
1	2 (25.0%)	2 (18.2%)
2	1 (12.5%)	1 (9.1%)
3	1 (12.5%)	1 (9.1%)
4	0 (0.0%)	0 (0.0%)
Unknown	4 (50.0%)	3 (27.3%)
Variables at decompression surgery		
Median age (range), years	72 (62–78)	65 (46–71)
Median prostate-specific antigen (range), ng/mL	910 (98–8900)	67 (0.1–307)
Lesion of decompression surgery, n (%)		
Cervical spine	0 (0.0%)	1 (9.1%)
Cervical-thoracic spine	0 (0.0%)	2 (18.2%)
Thoracic spine	6 (75.0%)	6 (54.5%)
Thoracic-lumbar spine	0 (0.0%)	1 (9.1%)
Lumbar spine	2 (25.0%)	1 (9.1%)
Median time to decompression surgery from symptoms occurrence (range), days	4 (0–8)	2 (0–14)
The use of bone-targeted agents^a^, n (%)	0 (0.0%)	4 (36.3%)

*mHNPC* Metastatic hormone-naïve prostate cancer, *mCRPC* Metastatic castration-resistant prostate cancer

^a^Zoledronic acid or denosumab

### Functional outcomes

Table 2 shows the functional outcomes evaluated by the Frankel grade before and after decompression surgery among men with mHNPC. Although two of eight patients (25.0%) with mHNPC were ambulatory (Frankel grade D and E) preoperatively, six patients (75.0%) were ambulatory postoperatively. Table 3 shows the functional outcomes evaluated by the Frankel grade before and after decompression surgery among men with mCRPC. Among 11 patients with mCRPC, only 3 (27.3%) were ambulatory preoperatively while 6 (54.5%) were ambulatory postoperatively.

**Table 2:** Surgery for metastatic spinal cord compression from metastatic hormone-naïve prostate cancer: functional outcome

FRANKEL Grade	Postoperative					Number of patients
Preoperative	A	B	C	D	E	Total (8)
A	1^a^					1
B		1^a^				1
C				1^b^	3^b^	4
D				2^c^		2
E						0

^a^These patients were nonambulatory both before and after decompression surgery

^b^These patients were nonambulatory before decompression surgery and ambulatory after surgery

^c^These patients were ambulatory both before and after decompression surgery

**Table 3:** Surgery for metastatic spinal cord compression from metastatic castration-resistant prostate cancer: functional outcome

FRANKEL Grade	Postoperative					Number of patients
Preoperative	A	B	C	D	E	Total (11)
A						0
B						0
C			5^a^	3^b^		8
D				2^c^	1^c^	3
E						0

^a^These patients were nonambulatory both before and after decompression surgery

^b^These patients were nonambulatory before decompression surgery and ambulatory after surgery

^c^These patients were ambulatory both before and after decompression surgery

### Oncological outcomes

**Fig. 1:**
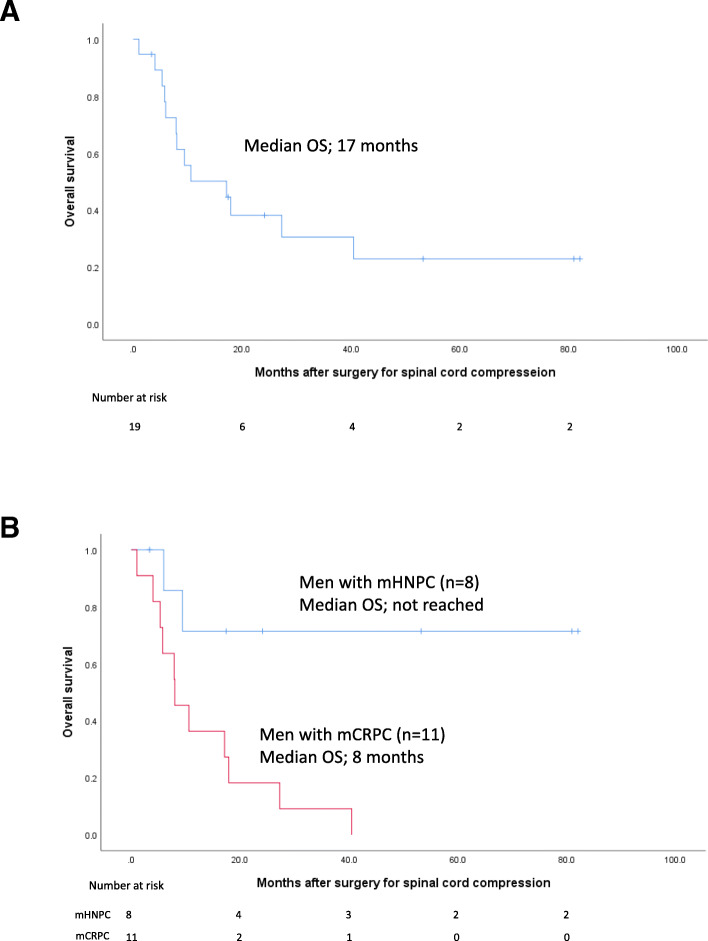
Kaplan–Meier curves for overall survival (OS). **a** Kaplan–Meier curve for OS among men treated with decompression surgery for metastatic spinal cord compression originating from prostate cancer in the whole cohort (*n* = 19). The median OS was 17 months. **b** Kaplan–Meier curve for OS among men treated with decompression surgery for metastatic spinal cord compression originating from metastatic hormone-naïve prostate cancer (mHNPC, *n* = 8) and metastatic castration-resistant prostate cancer (mCRPC, *n* = 11). The median OS after surgery was not reached in men with mHNPC and 8 months in men with mCRPC. The blue line indicates the survival of men with mHNPC, and the red line indicates the survival of men with mCRPC

Figure 1a shows the Kaplan–Meier curve for OS after decompression surgery in the entire cohort, and Fig. 1b shows the Kaplan–Meier curve among men with mHNPC and mCRPC. The median OS after decompression surgery in the entire cohort, men with mHNPC, and men with mCRPC was 17 months, not reached, and 8 months, respectively.

## Discussion

MSCC from PC may cause irreversible neurological impairment, gait disturbance, and a poor quality of life. Thus, MSCC is an oncologic emergency that requires accurate diagnosis and rapid treatment [14]. When considering the treatment strategy for symptomatic MSCC, multidisciplinary approaches are needed [14].

The definitive treatments for MSCC are surgery and radiation therapy. Postoperative rehabilitation also has an important role [15]. Patchell et al. [10] demonstrated that decompression surgery plus postoperative conventional external beam radiation was superior to treatment with radiation alone for MSCC from various cancers in a phase III randomized clinical trial. However, their study had some bias; a significant proportion of ambulatory patients underwent surgery, and nonambulatory patients were treated with radiotherapy [10]. Moreover, their study was published in 2005 [10]. PC treatment has been rapidly advancing; therefore, the efficacy of adding external beam radiation to decompression surgery should be re-evaluated in a prospective study.

The patient’s prognosis, general health status, neurologic/mechanical function, and surgical morbidity and mortality should be considered when determining the treatment strategy for MSCC [9, 14, 16, 17]. Several tools for predicting survival of men undergoing surgical treatment of MSCC have been reported [18]. A PC-specific prognostic nomogram for mHNPC [3] or mCRPC [19] is also useful for prediction of survival and can provide significant information to orthopedic surgeons when considering the indication for surgery and determining the optimal surgical procedure.

The preoperative neurological and mechanical functions are also important factors when considering the treatment strategy for MSCC. Clarke et al. [9] reported that only one patient with preoperative Frankel grade B had a good functional outcome after surgery, although most patients with preoperative Frankel grade C, D, or E showed a favorable postoperative functional outcome. In our study, all patients with Frankel grade A and B underwent surgery and showed an unfavorable functional outcome postoperatively. Although selection bias cannot be ruled out, preoperative Frankel grade A or B could be carefully considered as an indication for surgery.

In the present study, although two of eight patients with mHNPC were ambulatory preoperatively, six of eight patients were ambulatory postoperatively and the median survival after surgery for MSCC was not reached. Crnalic et al. [20] also reported that 13 patients treated with surgery for MSCC from mHNPC achieved long survival and that 9 of 12 patients were ambulatory postoperatively despite the fact that only 1 of the 12 patients had been ambulatory preoperatively. Recovery of gait function may contribute to improvement in the quality of life, especially in patients with a good prognosis. Although clinical evidence is scarce, surgery for MSCC in men with mHNPC appears to be beneficial in appropriately selected patients in terms of achieving both a favorable quality of life and a good prognosis.

Patients who underwent surgical treatment of MSCC from mCRPC had a median survival time of only 8 months after surgery in the present study. Crnalic et al. [20] also reported that 41 patients who underwent surgical treatment of MSCC from mCRPC had a median survival time of only 5 months. The survival benefit obtained from surgery for MSCC in patients with mCRPC might be low; however, these patients might be surgical candidates for a good quality of life in highly selected situations [9, 20, 21].

Our study has several limitations, including its retrospective design and small cohort. However, we demonstrated that decompression and reconstruction surgery for symptomatic MSCC from PC might contribute to a favorable functional outcome among men with mHNPC and mCRPC. However, its role in improving the oncological outcome remains unclear. The treatment strategy should be determined by shared decision-making among patients, urologists, radiation oncologists, and orthopedic surgeons.

## Conclusions

Our retrospective study has demonstrated that decompression and reconstruction surgery for symptomatic MSCC from PC might contribute to a favorable functional outcome among men with PC. Hormone-naïvety and mechanical/neurological function might be correlated with the postoperative outcome. The treatment strategy should be determined by shared decision-making among patients, urologists, radiation oncologists, and orthopedic surgeons.

## Data Availability

Because of ethical restrictions, the raw data underlying this paper are available upon request to the corresponding author.

## Abbreviations

MSCC: Metastatic spinal cord compression
PC: Prostate cancer
mHNPC: Metastatic hormone-naïve prostate cancer
mCRPC: Metastatic castration-resistant prostate cancer
OS: Overall survival
